# Ending the violent streak

**DOI:** 10.1016/j.lanwpc.2021.100364

**Published:** 2021-12-23

**

On November 26, the UN marked International Day for the Elimination of Violence Against Women with 16 days of activism. Violence against women is an abhorrent and prevalent health issue. Globally, WHO estimates that one in three women will experience intimate partner violence (IPV) or non-partner sexual violence in their lifetimes. Within the Western Pacific region, the lifetime prevalence of IPV in women aged 15–49 years in each country ranges from 11% to 53%. The high risk is particularly pronounced in the region's low-income and middle-income countries, with Fiji, Kiribati, Papua New Guinea, and the Solomon Islands reporting a prevalence of more than 50% lifetime risk of IPV. Reports of IPV have also increased five-fold during the COVID-19 pandemic, emphasising the critical need for domestic violence services and support.

Acts of violence or abuse are incredibly heterogenous and include physical, sexual, psychological, or financial violence. In 1993, the UN defined the term “violence against women” as “any act of gender-based violence that results in, or is likely to result in, physical, sexual, or mental harm or suffering to women, including threats of such acts, coercion or arbitrary deprivation of liberty, whether occurring in public or in private life”. Clear examples include battery, rape, or genital mutilation. However, many types of violence are harder to identify, especially during lockdowns as a result of the pandemic. These acts can consist of cyber harassment, unwanted sexual advances, or controlling behaviours such as limiting financial access or acts of isolating individuals away from support networks. Across the whole Western Pacific region, 20% of women are reported to experience lifetime IPV. However, these figures do not include the majority of the cases that go unnoticed or unreported due to cultural acceptance, patriarchal ideology, limited resources, or the unwillingness of providers to enforce the laws. Despite the complexity, all forms of violence have dire impacts on the lives of victims.

The impacts of violence against women are multifaceted and affect many parts of women's lives. Health consequences such as homicide and severe injuries are commonly reported in the media. However, the violence can also cause other health problems such as exposure to sexually transmitted diseases, unwanted pregnancies, depression, anxiety, and suicide. Secondary effects of violence and abuse include poor overall health, pain syndromes, and increased susceptibility to smoking or substance use. Women who experience abuse also face economic repercussions. A 2015 World Bank Report found that women who experienced IPV were more likely to be in part-time and vulnerable employment. Compared with women who had not experienced abuse, victims of gender-based violence earned 60% less in wages, which adds to the complexity of empowering women to leave abusive relationships in situations with substantial socioeconomic disparities.

Eliminating violence against women requires systematic changes and effort through all levels of society. To enable this goal, WHO and UN Women disseminated a framework in 2019 targeted at policy makers entitled “RESPECT Women”. The seven strategies of the framework are represented by each letter of RESPECT: relationship skills strengthening; empowerment of women; services ensured; poverty reduced; environments made safe; child and adolescent abuse prevented; and transformed attitudes, beliefs and norms. The framework includes concrete suggestions to enable change and evidence levels for each suggestion's effect in different country income settings. For example, there is strong evidence for the effectiveness of group-based workshops that promote gender equality ideology to strengthen relationship skills in low-income and middle-income countries. By contrast, there is strong evidence of efficacy for couples therapy in high-income countries. The inclusion of evidence levels will help policy makers tailor strategies to each country's unique circumstances.

Evidence-based frameworks are an excellent way to encourage systematic change, but they will not end the violence alone. Translation of these guidelines is crucial to enacting real differences in the lives of women who face abuse. We need organisations, big and small, to recognise their role in encouraging healthier attitudes, building protective environments, and developing supportive policies. These actions could include more mental health support for perpetrators to assist with rehabilitation or high-visibility public health campaigns to help the community recognise signs of violence. Training service providers (such as health-care professionals and enforcement officials) to recognise abuse and enforce policies will further help eliminate violence. We need changes in all levels of society, from leaders and policy makers to the citizens of our world, to commit to ending the abuse. The 16 days of activism for the Elimination of Violence against Women concluded on December 10. But to make a lasting impact on women's lives, efforts must be ongoing throughout the year. Only through systematic efforts in all levels of society can the acceptance of violence against women be broken. Violence is not inevitable, and working towards elimination will empower women, improve gender equality, and help us achieve the Sustainable Development Goals to ensure no one is left behind.

